# Enteric Fever Presenting With Complete Heart Block (CHB): A Rare Case of Reversible Arrhythmia

**DOI:** 10.7759/cureus.56121

**Published:** 2024-03-13

**Authors:** Rushikesh H Dhondge, Sourya Acharya, Sunil Kumar, Sachin Agrawal, Shubham V Nimkar

**Affiliations:** 1 General Medicine, Jawaharlal Institute of Postgraduate Medical Education and Research, Wardha, IND; 2 Medicine, Jawaharlal Nehru Medical College, Datta Meghe Institute of Medical Science (Deemed to be University), Wardha, IND

**Keywords:** arrhythmias, typhoid, myocarditis, cardiomyopathy, cardiovascular

## Abstract

*Salmonella enterica* serovar Typhi is the causative agent of enteric fever, commonly called "typhoid". This fever can be mistaken for a variety of other febrile disorders. It is an endemic sickness, especially in developing nations. Enteric fever typically manifests with fever, abdominal pain, and constitutional symptoms, making it a diagnostic challenge due to its broad clinical spectrum. Enteric fever also affects various other systems, causing complications, amongst which the cardiovascular system is no exception. Complications in the cardiovascular system may range from myocarditis to cardiomyopathy and various arrhythmias. This case report describes a case of a 28-year-old male who presented to us with fever and giddiness. Examination revealed profound bradycardia and electrocardiography (ECG) revealed features of a complete heart block (CHB). Investigations for fever confirmed enteric fever. This case report highlights one of the rarest complications of enteric fever.

## Introduction

The bacterium *Salmonella enterica* serotype Typhi, also referred to as *Salmonella* Typhi, is the cause of typhoid fever, or simply "typhoid" [1]. Typhoid fever and paratyphoid fever are both enteric fevers. *Salmonella enterica *Typhi is thought to infect and reproduce only within humans. Abdominal pain and a high temperature are among the systemic symptoms that define this enteric fever. When neglected, it can sometimes lead to effects that mostly impact the gastrointestinal system, which is the pathology's site. Rarely, because of its unusual presentation, it can also affect various other systems of organs and make diagnosis difficult.

Cardiovascular complications linked to enteric fever are cardiomyopathy, myocarditis, endocarditis, pericarditis, congestive heart failure, and arrhythmias. Among various arrhythmias, the Wenckebach phenomena [2] and ventricular bigeminy [3] have been reported in cases of typhoid. Complete heart block (CHB) is an uncommon presentation.

## Case presentation

**Figure 1:**
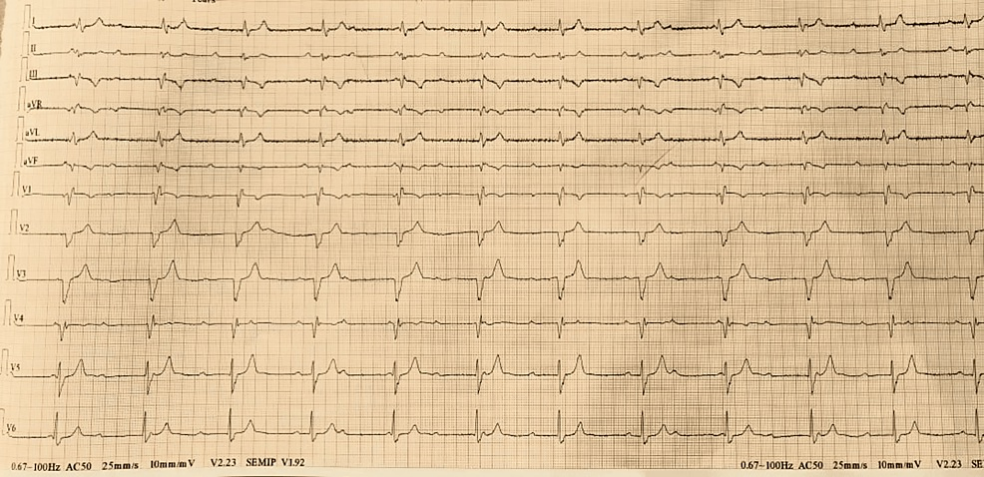
A 12-lead ECG of the patient showing CHB ECG: electrocardiogram; CHB: complete heart block

A 28-year-old male presented to the hospital with a one-week history of ongoing fever and three to four days of vomiting. The fever was characterized by its acute onset, high grade, and intermittent nature, peaking at 104°F. Notably, the patient did not experience rigors or chills after paracetamol administration. Additionally, he reported a constant bilateral headache, abdominal pain, and associated vomiting occurring three to five times a day. The abdominal content was watery with food particles, and the patient also complained of mild chest discomfort and giddiness. The patient had no history of systemic hypertension, diabetes mellitus, bronchial asthma, tuberculosis, and thyroid disorders. On admission, the patient was febrile and had a temperature of 104°F, bradycardia with a pulse rate of 42 beats per minute, blood pressure of 100/60 mmHg, and a respiratory rate of 16 per minute. Abdominal examination revealed mild tenderness without distension, while neurological and respiratory examinations showed no significant abnormalities. Cardiovascular system examination revealed a normal point of maximal impulse (PMI), and auscultation revealed variable intensity of S1. There were no murmurs, S3, S4, or rub. Electrocardiography was done, and it revealed atrioventricular (AV) dissociation with a constant R-R interval independent of P waves with a rate of approximately 42 beats per minute, suggestive of CHB (Figure 1).

Hemoglobin levels of 14 g/dL, total leukocyte counts of 2.6 x 109/L, platelet counts of 1.96 lakh/cumm, mean corpuscular volume (MCV) of 85 fL with neutrophils at 82% and lymphocytes at 15%, and an elevated erythrocyte sedimentation rate of 44 mm/h were all found in the laboratory test. In addition, serum urea: 24 mg/dL, serum creatinine: 1.1 mg/dL, serum sodium: 137 mmol/L, serum potassium: 4.8 mmol/L, serum calcium: 8.2 mmol/L, serum magnesium: 1.9 mmol/L, serum phosphorous: 4.2 mmol/L, aspartate aminotransferase: 49 U/L, alkaline phosphatase: 110 U/L, alanine aminotransferase: 39 U/L, total bilirubin: 1.1 mg/dL, total protein: 7.5 gm/dL, random blood sugar: 128 mg/dL, CPK-MB: 8 U/L, Trop-I: 1.5 pg/dL, and international normalized ratio: 1.2. Blood cultures were found to be positive for *Salmonella enterica* serotype paratyphi A, and the widal test was positive. The widal test showed *Salmonella* typhi O antigen titer of 160 and *Salmonella* H antigen titer of 200. Blood culture and sensitivity were done, and the patient was susceptible to all of the prevalent antibiotics (ampicillin, co-trimoxazole, gentamicin, amikacin, colistin, piperacillin, meropenem, chloramphenicol, tetracycline, ciprofloxacin, ofloxacin, ceftizoxime, cefotaxime, and ceftriaxone).

The patient received intravenous fluid resuscitation upon admission to the medical inpatient facility, as well as the initiation of injectable ceftriaxone (2 g) 12 hourly for 14 days. A cardiology consultation was done which suggested observation in ICU.

On the third day, the fever subsided. On the fourth day, his ECG reverted to normal sinus rhythm. The patient was kept on injectable ceftriaxone for 14 days and was discharged. On follow-up after a week, he was asymptomatic and had a normal sinus rhythm.

**Figure 2:**
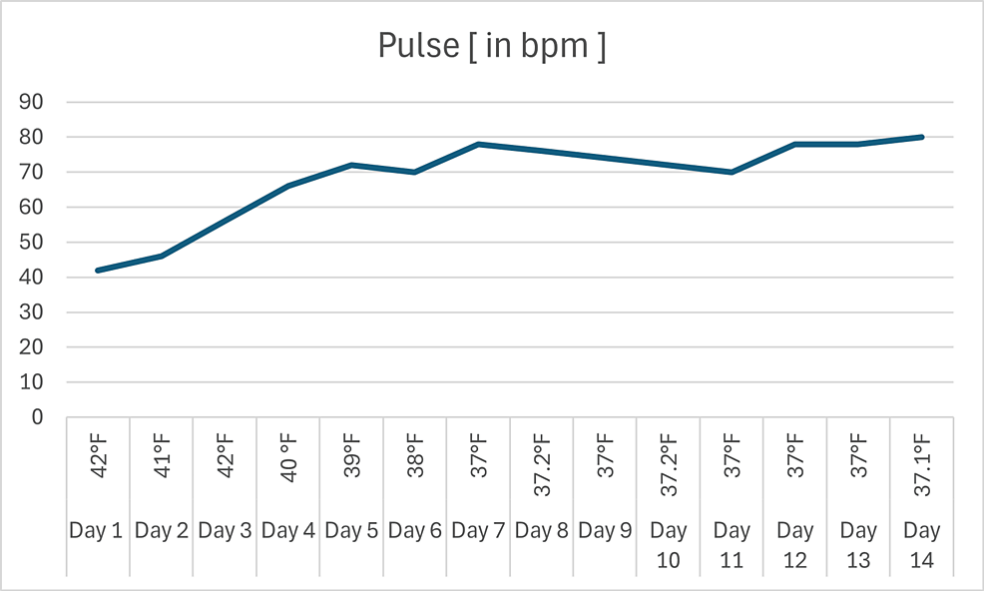
Step-ladder fever pattern of the patient along with the pulse rate bpm: beats per minute

The step-ladder fever pattern of the patient along with the pulse rate is shown in Figure 2.

Blood tests and diagnostic work-up of the patient are given in Table 1.

**Table 1:** Lab investigations

Lab Parameters	Observed Value	Normal Range
Hemoglobin	14	13-17 gm%
Total leucocyte count	2600	4000-11000/cumm
Platelets	1.96	1.5-4 lakh/cumm
Mean corpuscular volume	85	83-101 fL
Urea	24	19-43 mg/dL
Serum creatinine	1.1	0.66-1.25 mg/dL
Potassium	4.8	3.5-5.1 mmol/L
Sodium	137	137-145 mmol/L
Aspartate aminotransferase	79	Male < 17-59 U/L, Female 14-56 U/L
Alkaline phosphatase	110	38-126 IU/L
Alanine aminotransferase	39	Male < 50 U/L, Female < 35 U/L
Total protein	7.5	6.3-8.2 gm/dL
Total bilirubin	1.1	0.2-1.3 mg/dL
Random blood sugar	128	140-200 mg/dL
Creatinine kinase myoglobin binding	8	9-16
Troponin I	1.5	Male: up to 13 pg/dL, Female: up to 9 pg/dL
Serum calcium	8.2	8.4-10.2 mg/dL
Serum magnesium	1.9	1.6-2.3 mg/dL
Serum phosphorous	4.2	2.5-4.5 mg/dL
International normalized ratio	1.2	1-1.3

## Discussion

Enteric fever, including typhoid and paratyphoid infections, remains a global health concern, causing over 26 million cases and 215,000 deaths annually. Although the United States reports fewer cases, the incidence is higher in developing countries, particularly in south-central Asia and southern Africa. Travelers to endemic regions and those with less access to vaccination and pretravel consultation face heightened risks. Factors such as sanitation, sewage, and water treatment systems influence the prevalence of typhoid fever. More people contract *Salmonella* typhi than *Salmonella* paratyphi. Notwithstanding the difficulties brought on by rising multidrug resistance, death rates have declined in response to improvements in research, treatment techniques, and novel medications, despite an increase in cases globally. In the era of routine antibiotic use, classic manifestations including splenomegaly and rose spots are less commonly recognized [4]. 

The first and second weeks are when complications frequently occur. Common ECG abnormalities associated with enteric fever include QTc interval prolongation (caused by tissue anoxia-induced potassium (K+) loss from myocardial cells), decreased amplitude of QRS complexes (perhaps as a result of myocarditis), alterations to the ST-T segment, PR interval prolongation, right bundle branch block and Wenckebach phenomenon [2], and ventricular bigeminy [3]. Ventricular ectopics in myocarditis patients have rarely been reported. However, a rare ECG finding with enteric fever is CHB. Isolated ECG abnormalities are found in 40-80% of cases. Except for bundle branch block, which may last longer, ECG abnormalities occur during the peak of the pyrexial sickness and are temporary. Cardiovascular complications, though occurring in only 1-5% of patients, encompass a spectrum of myocarditis and endocarditis, with heart blocks being a rare but recognized association [5].

Untreated typhoid can cause extra-intestinal issues that impact the pulmonary, rheumatological, central neurological, and hepatobiliary systems, in addition to gastrointestinal symptoms.

Enteric fever can also affect the pancreas, leading to pancreatitis. It is thought that direct pancreatic infestation of the major pancreatic duct causes acute pancreatitis in cases of enteric fever. This could occur especially in scenarios when there are risk factors for biliary stasis, such as cholelithiasis, choledocholithiasis, and anomalies in the bile channel. On the other hand, our patient showed no signs of bile duct or gallbladder disease. Pancreatitis induced by the localized strain of *Salmonella* usually arises from choleraesuis-related *Salmonella* bacteremia, although it can also follow gastroenteritis from *Salmonella* typhimurium and enteric fever from *Salmonella* typhi. Pancreatitis can be accompanied by the emergence of a pancreatic abscess [6].

Myocarditis is a rare occurrence in typhoid, with only a handful of cases reported to date, dating back to the late 19^th^ century. Its presentation may mimic myocardial ischemia, or it can remain asymptomatic before causing sudden, nonspecific hemodynamic deterioration [7]

The contractions of the ventricles and atria are not synchronized, and a third-degree AV block indicates a total loss of communication between them. Due to the sinoatrial (SA) node's inability to regulate heart rate and its lack of proper conduction through the AV node, there is a reduction in cardiac output. There are several different circumstances that might lead to AV blocks, including third-degree blocks. Among these include idiopathic fibrosis, medication toxicity, nodal ablation, structural heart disease, acute ischemic heart disease, electrolyte abnormalities, and post-operative heart block following transcatheter or surgical aortic valve replacement. Other etiologies include systemic lupus erythematosus, sarcoidosis, amyloidosis, and collagen vascular diseases, in addition to Lyme disease [8].

In summary, typhoid-associated CHB is a rare but serious complication requiring a multidisciplinary approach. The understanding of its pathophysiology and appropriate treatment strategies are crucial for optimal patient outcomes.

## Conclusions

This case underscores the complexity of enteric fever, its varied clinical presentation, and the rare but potentially severe cardiac complications associated with *Salmonella* typhi infection. The comprehensive approach included prompt diagnosis, antibiotic therapy, and intensive care management. One important thing to keep in mind is that the majority of arrhythmias due to enteric fever are self-limiting and reversible. Regular follow-ups emphasize the importance of monitoring and assessing the long-term cardiac impact of such complications.
